# Live Fast, Die Young: Experimental Evidence of Population Extinction Risk due to Climate Change

**DOI:** 10.1371/journal.pbio.1002281

**Published:** 2015-10-26

**Authors:** Elvire Bestion, Aimeric Teyssier, Murielle Richard, Jean Clobert, Julien Cote

**Affiliations:** 1 CNRS USR 2936, Station d'Ecologie Expérimentale de Moulis, Moulis, France; 2 CNRS, Université Toulouse III Paul Sabatier, ENFA; UMR5174 EDB (Laboratoire Évolution & Diversité Biologique), Toulouse, France; 3 Environmental and Sustainability Institute, College of Life and Environmental Sciences, University of Exeter, Penryn, United Kingdom; 4 Terrestrial Ecology Unit, Ghent University, Ghent, Belgium; University College London, UNITED KINGDOM

## Abstract

Evidence has accumulated in recent decades on the drastic impact of climate change on biodiversity. Warming temperatures have induced changes in species physiology, phenology, and have decreased body size. Such modifications can impact population dynamics and could lead to changes in life cycle and demography. More specifically, conceptual frameworks predict that global warming will severely threaten tropical ectotherms while temperate ectotherms should resist or even benefit from higher temperatures. However, experimental studies measuring the impacts of future warming trends on temperate ectotherms' life cycle and population persistence are lacking. Here we investigate the impacts of future climates on a model vertebrate ectotherm species using a large-scale warming experiment. We manipulated climatic conditions in 18 seminatural populations over two years to obtain a present climate treatment and a warm climate treatment matching IPCC predictions for future climate. Warmer temperatures caused a faster body growth, an earlier reproductive onset, and an increased voltinism, leading to a highly accelerated life cycle but also to a decrease in adult survival. A matrix population model predicts that warm climate populations in our experiment should go extinct in around 20 y. Comparing our experimental climatic conditions to conditions encountered by populations across Europe, we suggest that warming climates should threaten a significant number of populations at the southern range of the distribution. Our findings stress the importance of experimental approaches on the entire life cycle to more accurately predict population and species persistence in future climates.

## Abstract 2

Warmer climates accelerate the pace of life of lizards and this demographic change leads to a strong decrease in population growth rate that may ultimately result in population extinctions.

## Author Summary

Ongoing climate change has potentially drastic impacts on biodiversity. Because their body temperature depends on their external environment, ectotherm (“cold-blooded”) species are thought to be more at risk from warming climates than endotherm (“warm-blooded”) species that regulate their temperature internally. Tropical ectotherms should be particularly threatened by climate change, while temperate ectotherms should resist or even benefit from higher temperatures. While most of the evidence on the impacts of climate change comes from long-term field studies, experimental evidence of the impact of future climatic conditions is still lacking. Here we investigate the impacts of future climates on a temperate lizard using a seminatural warming experiment. We find that warmer temperatures led to a highly accelerated life cycle and a decrease in adult survival. As a result, we postulate that populations in such warm climates would be expected to go extinct in around 20 y. Comparing our experimental conditions to climatic conditions in European populations of common lizards, we show that many populations should be threatened in the next century, particularly in Southern Europe. Our findings challenge the optimistic view that climate change is only a threat for tropical ectotherms and stress the importance of experimental approaches to predicting the consequences of future warming trends.

## Introduction

Over the last decades, consequences of global warming on biodiversity have become obvious [1–3], with many species likely to be committed to extinction by 2050 [4]. Climate warming has already led to changes in species phenology [1], physiology (increased metabolic rates [5]), morphology (shrinking body size [6]), life cycle demography [7], and distribution [1], and, as a consequence, in community structure [8]. Because their body temperature, and hence their basic physiological functions, directly depend on environmental conditions, ectotherms are particularly at risk with climate change [5], while the number of studies assessing their response to changing climate is far lower than for endotherms [9]. The evaluation of their vulnerability is therefore urgent. For instance, a recent study predicted local extinctions of populations from various lizard families worldwide to reach 39% by 2080 due to climate change [10]. Theoretical studies predict that climate change will principally threaten tropical ectotherms [11–14], while temperate ectotherms should resist or even benefit from the warmer temperatures [13,15–17]. However, most evidence on the impacts of climate change on species comes from long-term field survey data [1,8], or on the contrary, on short term laboratory experiments lacking ecological realism and complexity [18–20]. Despite the growing evidence on the strong impact of ecological context on species adaptation to temperature [21], there is little large scale realistic experimental evidence on animals, especially on vertebrates [20,22–25]. More importantly, to our knowledge, the impact of climate change on a species’ entire life cycle and population persistence has never been experimentally tested on a vertebrate [26]. This information gap hinders the prediction of future impacts, because unraveling the impact of predicted climate on different demographic parameters is essential for the precise estimation of extinction probability [27,28]. The Intergovernmental Panel on Climate Change (IPCC) predicts a global temperature increase between +0.3 and +4.8°C over the next century, depending on the CO_2_ emission scenarios [29]. Experimental studies should thus implement realistic IPCC climate change projections relying on several greenhouse gas emission scenarios and describe population responses to said scenarios in large field experiments [24,25].

**Fig 1:**
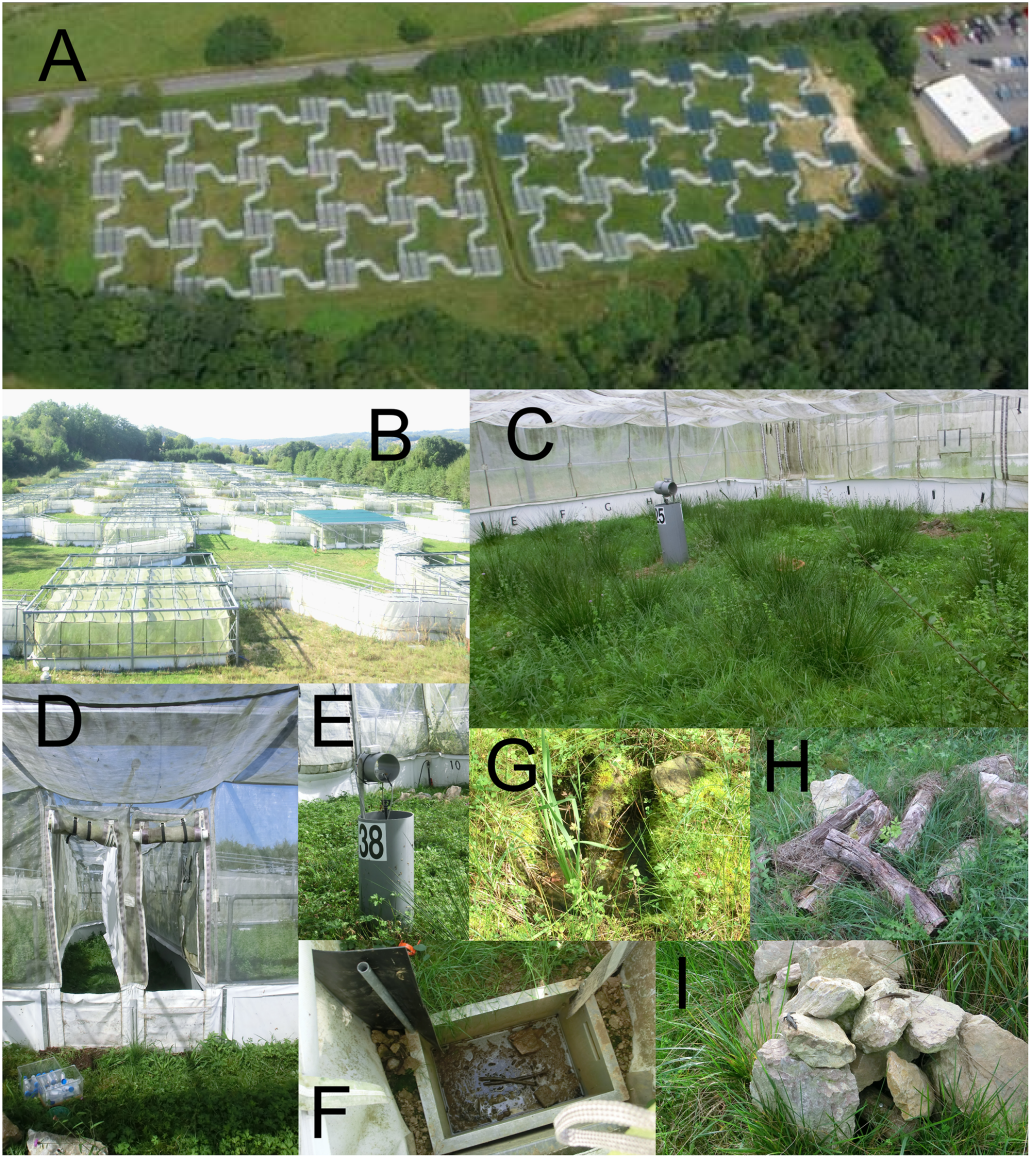
The Metatron. **A**: Aerial view of the structure. On the right, top shutters are closed on 17 enclosures. Credits: Quentin Bénard. **B**: Close view of the structure. On the bottom left, an enclosure with open shutters. On the top right, an enclosure with closed shutters. **C**: Inside view of one enclosure. **D**: Entrance of the two half-corridors of one enclosure. **E**: Pole containing the sensors recording temperature, humidity, and illuminance inside of the enclosure as well as the sprinkler system, protected with plastic and labeled with the patch identification number. **F**: Pitfall trap at the end of one corridor. **G**: One of the two ponds set in each enclosure. **H** and **I**: Rock and logs allowing for lizard thermoregulation, set in each corner of the enclosures.

Here, we studied the effect of a warmer climate on the life cycle and demography of a lizard species with large-scale experimental mesocosms (Fig 1). Using common lizards (*Zootoca vivipara*) as model species, we aimed to determine whether predicted temperature increases will be detrimental or beneficial to temperate lizards and to identify the key parameters involved in potential declines of populations, especially in populations at the southern margin of the distribution area. To that end, we took advantage of an innovative experimental facility, the Metatron, a system with large seminatural enclosures in which climatic conditions can be manipulated (Fig 1) [30].

We created 18 lizard populations in the Metatron over two years of experiment (2012: 8 populations, 2013: 10 populations) and allocated them to one of two climatic treatments throughout the summer: “present climate” (existing local area climate) and “warm climate” (~2°C warmer than ambient temperatures), coherent with IPCC climate change projections for the end of the century (global temperature increase projections for a midrange emission scenario, Representative Concentration Pathway (RCP) 4.5: +1.8 ± 0.5°C [29]). We investigated adult and juvenile survival, body growth, and female reproduction to estimate the effect of warmer climatic conditions on lizard life history and population growth rate. We further compared our results to climatic conditions across Europe to inform predictions about more general fate of European lizard populations.

## Results

### Impact of Climate Change on Juveniles

**Fig 2:**
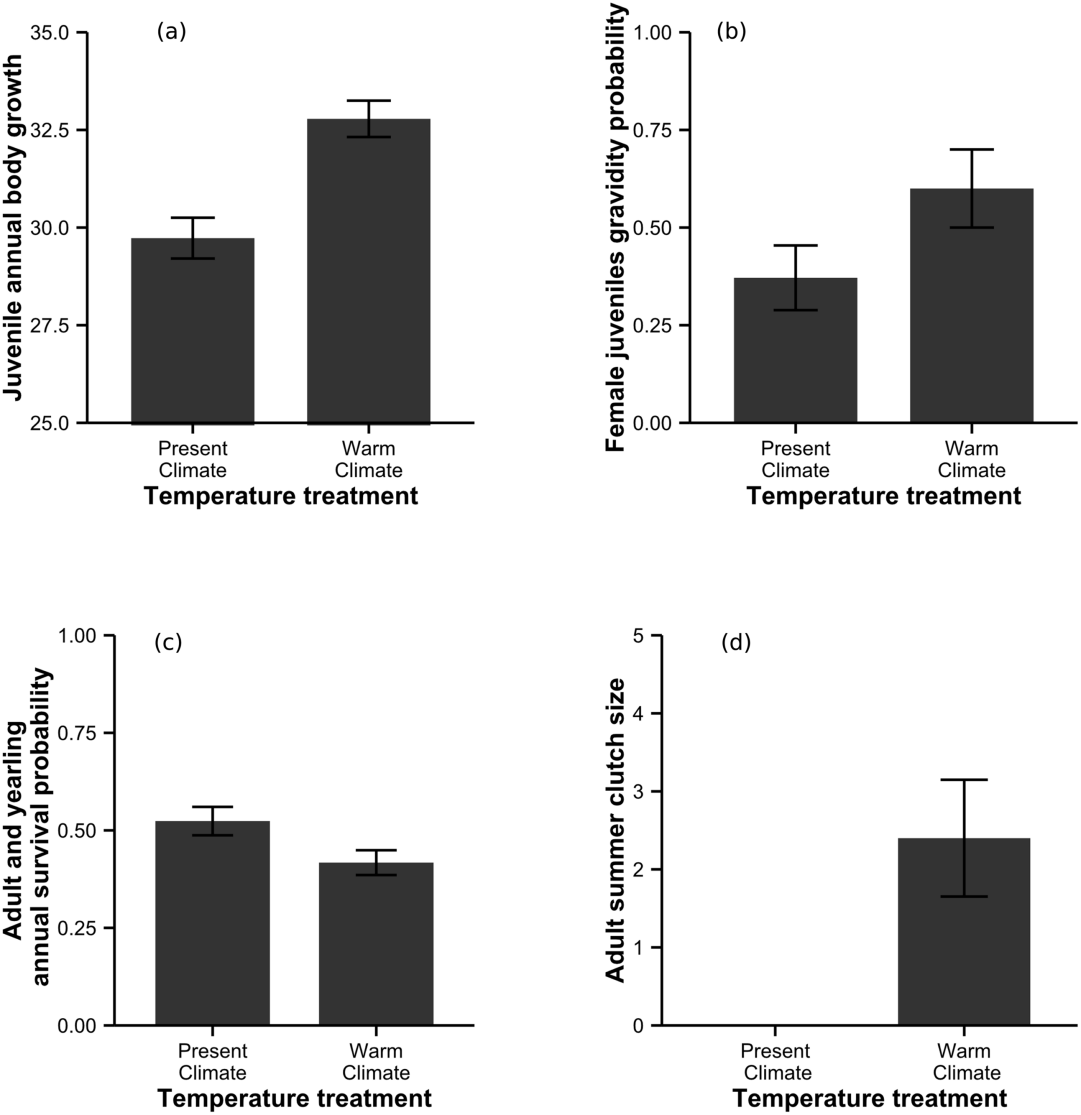
**(a)** Juvenile annual body growth (mean ± SE) depending on the temperature treatment. Body growth is calculated as the difference between snout–vent length at birth and snout–vent length at recapture after one year, measured in mm. **(b)** Female juvenile probability of gravidity at one year old (mean ± SE) depending on the temperature treatment. **(c)** Adult and yearling annual survival probability (mean ± Standard Error [SE]) depending on the temperature treatment. **(d)** Clutch size of females that laid a second clutch during the 2012 summer (mean ± SE) depending on the temperature treatment. Underlying data can be found in S7 Table.

Warm climatic conditions had a strong positive impact on juvenile body growth (Table 1, Fig 2a) but had no effect on body condition (Table 1). Warm climates also led to an earlier reproductive onset in these juveniles. Indeed, female juveniles from the “warm summer climate” populations were more likely to reproduce the following spring (Table 1, Fig 2b). This accelerated reproductive onset was likely due to the higher individual body growth rate, as female body size in May had a significant impact on probability of gravidity (Likelihood ratio test, χ² = 24.9, *p* < 0.001). There was no overall effect of climate treatment on annual survival (Table 1), although juveniles from the “warm climate” treatment tended to survive less during the summer (S3 Table).

**Table 1:** Effect of temperature treatment on survival, body growth, body condition, and female reproduction the following year in juveniles.

	Best Model	Likelihood ratio test (df = 1)		Effect of the temperature treatment		Effect of the date of birth		Effect of sex		R²		Proportion change in variance (PCV)			
		χ²	*p*	Estimate	SE	Estimate	SE	Estimate	SE	R²m	R²c	enclosure	family	year	residuals
Annual survival	BirthDate + (1|Enclosure) + (1|Family)	2.69	0.101			0.019	0.013			0.008	0.206	−0.006	−0.01		
Annual body growth	**Temp** + BirthDate + (1|Enclosure)	12.16	<0.001 ***	2.84	0.79	−0.14	0.03			0.186	0.434	0.193			0.193
Spring body condition	BirthDate + (1|Family)	0.96	0.326			−0.003	0.003			0.015	0.214		0.06		0.003
Probability of gravidity (t + 1)	**Temp** + BirthDate + (1|Enclosure)	4.53	0.033 *	1.33	0.62	−0.05	0.03			0.114	0.114	1			
Clutch size (t + 1)	BirthDate + (1|Year)	1.85	0.174			0.001	0.01			0.0002	0.009				0
Laying date (t + 1)	BirthDate + (1|Family)	0.1	0.757			0.34	0.18			0.127	0.869		0.12		0.075

NOTE: Statistics of Likelihood Ratio test compare two models, one with temperature treatment and one simpler model without temperature treatment. Generalized linear mixed models with logit links are used for binomial factors such as survival and gravidity, other variables are modeled with linear mixed models except for clutch size, which is modeled with a Poisson distribution. We provide estimate and standard error of the fixed effects included in the model (temperature treatment and date of birth). Following Nakagawa and Schielzeth (2013), we also provide marginal (R²m, effect of the fixed effects) and conditional (R²c, effect of the fixed and random effects) R² for our best models, as well as the proportion change in variance (PCV) for the random effects.

### Impact of Climate Change on Adults and Yearlings

Warmer climate was detrimental for the survival of older individuals. The annual survival of adults and yearlings was lower in “warm climate” environments (Table 2, Fig 2c), and this effect was mainly due to a difference of survival during the summer (S3 Table). Warmer climatic conditions had, however, a positive impact on the body condition of adults that survived (Table 2), while there was no impact of climatic conditions on individual growth rate. We further found a tendency for an earlier laying date in adult females from the “warm climate” enclosures (Table 2). Moreover, we found out that some females had produced a second clutch during the summer 2012. Twelve neonates, from five females, hatched in the “warm climate” enclosures during the summer (Fig 2d), while we did not find such neonates in “present climate.” These neonates were born from a second clutch of these females.

**Table 2:** Effect of temperature treatment on survival, body growth, body condition, and female reproduction the following year in yearling and adults.

	Best Model	Likelihood ratio test (df = 1)		Effect of the temperature treatment		Effect of age		Effect of sex		R²		Proportion change in variance (PCV)			
		χ²	p	Estimate	SE	Estimate	SE	Estimate	SE	R²m	R²c	enclosure	family	year	residuals
Annual survival	**Temp** + age + sex + (1|Enclosure)	3.92	0.048 *	−0.55	0.28	−0.58	0.22	0.65	0.21	0.058	0.147	0.134			
Annual body growth	Age + sex + (1|Year)	0.25	0.617			6.75	0.44	0.07	0.4	0.389	0.686			0.1	0.553
Spring body condition	**Temp** + age + sex + (1|Year)	5.86	0.015 *	0.23	0.09	−0.12	0.11	0.16	0.1	0.045	0.089			−0.33	0.074
Probability of gravidity (t + 1)	Age + (1|Enclosure)	0.35	0.553			0.05	0.24			0.0001	0.0001				
Clutch size (t + 1)	Age + (1|Year)	0.26	0.613			−0.09	0.1			0.009	0.02			0.186	0
Laying date (t + 1)	**Temp** + age + (1|Enclosure) + (1|Year)	3.1	0.078	−4.11	2.2	2.12	2.03			0.031	0.565	0.333		−0.03	0.015

NOTE: Statistics of Likelihood Ratio test compare two models, one with temperature treatment and one simpler model without temperature treatment. Generalized linear mixed models with logit links are used for binomial factors such as survival and gravidity, other variables are modeled with linear mixed models except for clutch size, which is modeled with a Poisson distribution. We provide estimate and standard error of the fixed effects included in the model (temperature treatment, age, and sex). Following Nakagawa and Schielzeth (2013), we also provide marginal (R²m, effect of the fixed effects) and conditional (R²c, effect of the fixed and random effects) R² for our best models, as well as the proportion change in variance (PCV) for the random effects.

### Population Growth Rate

We modeled the impact of our climatic treatment on lizard population dynamics with an age-structured Leslie matrix fitted with the survival and reproduction parameters obtained from our field experiment (S3 Text, S3 Fig, S4 Table). Population growth rate in “warm climate” environments was very low (λ = 0.75 [0.72, 0.77], mean [95% CI], results for a deterministic model), while populations in “present climate” environments were maintaining themselves (λ = 0.98 [0.95, 1.01], confidence interval crossing 1). As a consequence, populations in warm climates should go extinct rapidly (years to extinction, mean [95% CI], warm climate = 22 y [20, 24], present climate = 298 y [118, no extinction]). Using a stochastic model yielded very similar results (S3 Text).

### Consequences for European Populations

**Fig 3:**
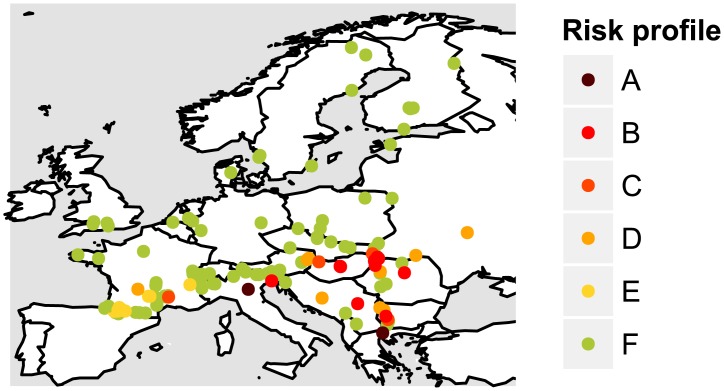
Potential risk from climate change for common lizard populations across Europe inferred from current maximum temperatures experienced by these populations. Colors represent “risk profiles” of the populations, from A: imminent risk (purple) to F: low risk (green), see S4 Text, S6 Table. Populations in risk levels from A to C (purple, red and dark orange) will be threatened by a 2°C increase in temperatures. Populations in risk level D (light orange) will be threatened by a 3°C temperature increase, and risk level E (yellow) will be threatened by a 4°C temperature increase.

We compared maximum daily temperatures in common lizard populations across Europe to maximum daily temperatures experienced by lizards in our experimental setup to categorize populations into “risk profiles” (S4 Text, S6 Table). We showed that under a 2°C temperature increase scenario, a significant number of European populations, mostly at the southern margin of the distribution, may be at risk from warming climates. Fourteen percent of European populations may be threatened in the future if temperature increases by 2°C (Fig 3, S4 Text, risk levels A to C). Moreover, if temperature rises by 3°C, 21% of the populations might be at risk in the future (Fig 3, S4 Text, risk levels A to D). Additionally, comparing with a survey done by Sinervo et al. [10] on European populations of common lizards, we found that populations classified by the authors as nearly extinct or extinct fell significantly more within our “at risk” profiles than populations classified as maintaining themselves (χ² = 7.8, *p* = 0.005, S4 Text). Risk profile projections depend on the demographic parameters obtained from our experiment, and as such should be sensitive to differences in demographic parameter estimates in the natural populations, particularly on changes in adult and juvenile survival rates (S3 Text, S5 Table), as well as on uncertainty in climatic data observations.

## Discussion

We found that warmer climatic conditions strongly modified lizard's life history. On one hand, warm climatic conditions had a strong positive impact on juvenile body growth. In ectotherms, a difference of 2°C, as generated in our experiment, can largely increase metabolic rate [5] and hence energetic needs. When juveniles can compensate for this increased metabolism by foraging more, it should lead to a faster body growth rate. Invertebrate diversity and abundance were high in enclosures, and there was no difference between climatic conditions (*p* > 0.55, S1 Text, Material and Methods). Juveniles could thus compensate by foraging more, resulting into a faster growth rate with subsequent consequences on their entire life history. For instance, reproduction is size-dependent in reptiles [31] and should be favored by an accelerated individual growth. Indeed, female juveniles from the “warm climate” populations were more likely to reproduce the following spring because of the fast summer individual growth rate. Such results are consistent with patterns observed in natural populations, as body size and individual growth rate were shown to increase with temperature in common lizard populations [32–35], while age at first reproduction depended on body size [36]. Warm climatic conditions were therefore mostly beneficial at juvenile stages as juvenile survival during the summer was only slightly decreased in our experimentally warmer climates, with overall 30% of juveniles from all populations surviving their first year, as in natural populations [33,37].

On the other hand, a warmer climate was mostly detrimental for older individuals. Only 42% of adults and yearlings from the “warm climate” treatment survived after one year, while 52% survived in present climates, comparable to survival rates found in natural populations from France, Belgium, and the Netherlands [33,37–39]. One explanation for this difference could be a summer heat stress, daily temperatures surpassing lizard critical thermal maximum. However, this view was not supported, as temperature only rarely surpassed critical thermal maximum (CTmax = 40°C [40]), and as a large temporal and spatial thermal heterogeneity within enclosures allowed lizards to find cool conditions during warm hours in both climates (S2 Text, S1 Table). In addition, climatic conditions had no effect on juvenile survival, while juvenile individuals likely have lower CTmax, as in other lizard species [41,42]. A second, more likely hypothesis could be linked to metabolic costs [43]. In ectotherms, metabolic rate scales positively with body size and temperature [44]. Warmer temperatures should increase energetic needs that cannot be fully compensated by an increase in foraging, in particular when warming induces restriction of lizard activity period, as suggested by a recent study [10] (but see [45]). This explanation may also explain the discrepancy of effects between ages as the rise in energetic needs in smaller individuals (i.e., juveniles) may be more easily compensated by foraging. However, adult body condition did not decrease in warmer conditions over the summer (S3 Table) and even increased after the winter in surviving individuals from the warm climate (Table 2). As the better spring body condition can be explained by a lower lizard density and thus competition for food during the spring (impact of lizard density on adult body condition, Likelihood ratio test, χ² = 5.91, *p* = 0.02), our energetic needs hypothesis may still explain our results and would concur with previous results on fish [46,47] and marine invertebrates [48]. In these studies, juveniles and smaller individuals survived better in higher temperatures than larger ones, which were failing to meet overall energy demands [46]. On top of energy demands, a warming-accelerated metabolism and foraging could change various physiological parameters (e.g., increased oxidative stress [49,50]) leading to physiological exhaustion and mortality in adults only [50,51]. A last possibility is that our climatic treatments, mainly set in the summer, generated a mismatch between summer and winter temperatures, hence increasing mortality during the winter. Because adult mortality during the summer was already affected by climatic treatment (S3 Table), it seems however unlikely that a temperature mismatch between summer and winter temperature could be the sole cause of the observed mortality increase.

These negative impacts of a warmer summer climate on adult life expectancy could be balanced by a higher investment in reproduction. In this species, reproduction occurs once a year in the spring, but summer climatic conditions could change reproductive investment during the following spring. Although this change was not observed, we found out some females produced a second clutch during the summer of climate manipulation. Twelve juveniles, from five females, hatched in the “warm climate” enclosures during the summer 2012, while we did not find any neonates in “present climate.” These findings are surprising, as in natural populations viviparous common lizards have never been observed to reproduce twice a year [37,39], although oviparous common lizards can produce second clutches [36]. Increased voltinism due to climate warming has been recently demonstrated in butterflies [52], and, in multivoltine lizards (*Uta stansburiana*), bivoltinism frequency was shown to increase with nocturnal temperature [53]. However, this is the first study to our knowledge showing that a univoltine vertebrate can shift to multivoltinism due to environmental conditions. Nevertheless, second clutches were too rare to balance the drop in survival rate (S3 Text). Together with an earlier onset of reproduction and a decrease in adult survival, these results suggest an acceleration of common lizard population turnover as a response to climate warming. Theoretical studies demonstrate that warming can accelerate metabolic and demographic rates in ectotherms [3]. Our work provides the first experimental evidence of such demographic acceleration, which should in turn change population dynamics and persistence [54]. Indeed, the earlier onset of reproduction of young females in warmer conditions was not sufficient to compensate for the drop in adult and yearling survival at these temperatures. As population growth rate was more sensitive to survival rates than to yearling fecundity (S3 Text, S5 Table), populations in a warm climate were predicted to go extinct in around 20 y, while populations in a present climate maintained themselves (λ = 0.98, 95% CI for λ crossing 1, [0.95,1.01]). These predictions are made even worse by the absence of warming enhanced dispersal movements (S3 Table), which could allow individuals to track their climatic niche [55], but see [56].

When we compared climatic conditions in our experiment to conditions encountered by common lizard populations across Europe, we found that several populations at the southern margin of the distribution should be at risk from climate warming in the near future, while populations at the northern margin should not be threatened (Fig 3, S4 Text). Considering a scenario of around +2°C temperature increase by the end of the century (consistent with RCP 4.5 greenhouse gas emission scenario [29]), we showed that 14% of populations surveyed should be threatened by the climate change in the next century, 11% in the very near future (around 2050, S4 Text). If we consider a higher temperature increase of 3°C, which could be attained with RCP 6.0 high emission scenario, the percentage of threatened populations went up to 21%, and with a very high temperature increase scenario of 4°C, possible under RCP 8.5 emission scenario, it attained 30% of the populations (S4 Text). Moreover, we showed that two European populations, located at the extreme southern margin of the distribution, might already be threatened under the current levels of temperature (S4 Text, “imminent risk” profiles). Finally, we found that nearly extinct or extinct populations from Sinervo et al. survey [10] were more likely to fall within our “at risk with 2°C increase” profiles than populations found to maintain themselves, confirming that temperature was probably one of the main drivers of the observed extinctions. Further modeling on range dynamics and extinction risks of *Z*. *vivipara* and other lizard species should use spatially explicit demographic models (e.g., [28,57,58], but see [59] for a review of available methods) informed by our experimental results as well as by data from field surveys (e.g., density and demographic parameters), to draw a better picture of the impacts of climate change on lizard population and range dynamics under several greenhouse gas emission scenarios. Overall, we showed that lizard populations at the southern margin of their distribution should be particularly sensitive to a warming climate, leading to potential population extirpations and a shrinking of lizard’s range, while populations at higher latitudes should not be threatened. The limitation of a species range has been attributed to two interacting factors, abiotic conditions such as temperature and hygrometry and biotic conditions such as competitive interactions [60,61]. Our results support the idea that common lizards range is limited in the south by abiotic conditions due to the climate-dependent species demography.

Our study demonstrates for the first time a change in life history tactics due to a 2°C climate warming, with an acceleration of the pace of life and generation turnover. This acceleration was associated with a decrease in population density, which could lead to the extinction of common lizard populations at the southern margin of their distribution. Previous studies on natural populations of common lizards showed that the current rate of warming had rather positive effects on populations [32,35], mostly because they found either no effect or positive effects of warmer spring temperature over the past 20 y on body growth rate and/or survival, with the exception of one study showing slightly negative relationships between temperature and survival in some populations [38]. However, the effect of temperature is unlikely to be linear, and thermal physiology of ectotherm species suggests a threshold of temperature above which performance decreases steeply [62]. Our simulated warming matches the summer temperatures predicted for the end of the century and could exceed a threshold where thermal conditions shift from beneficial to detrimental for adult survival. If the trend of temperature increase follows IPCC predictions, we can predict demographic accelerations in ectotherm species. The functioning of communities strongly depends on the fine tuning of species interactions, and changes in species pace of life can destabilize community assemblages and hence induce their extinction [8,63]. Using a model species, our findings emphasize that climate change is not only a problem for tropical ectotherms [11–14] but, contrary to more optimistic predictions [13,15–17], it could endanger temperate ectotherms with population extirpations and a shrinking of their range of distribution by the disappearance of southern populations. In species with a restricted range distribution, such population extirpations could ultimately lead to species extinctions if these species are unable to adapt to warmer climates. Now, we should therefore study how species can adapt to future climatic conditions through phenotypic and phenological modifications. For instance, a selection for an earlier onset of reproduction and an increased voltinism might allow species to shift towards a faster life history strategy and populations to be rescued by compensating lower adult survival rates [64–66]. However, such acceleration of population turnover might, on the contrary, be detrimental. For instance, in a European butterfly (*Lasiommata megera*), an increased voltinism led populations into a developmental trap where individuals attempted third generations, resulting in higher mortality and the loss of the third generation [67]. Future experiments should therefore simulate future warmer climates on several generations to study species adaptiveness and persistence.

## Materials and Methods

The use of animals in this study was approved by the French Government, License no.2010-189-16 DREAL.

### Species and Experimental System

The common lizard (*Z*. *vivipara*; Jacquin 1787) is a small (adult snout–vent length 50–70 mm) viviparous lacertid lizard inhabiting dense vegetation patches across Europe and Asia. Common lizards have been extensively studied for their biology and population dynamics (e.g., [32,35–37,68–70], S6 Table), making them a good model species to study the consequences of climate change on temperate lizards. Lizards hibernate from October to March in our study site (Ariège, France), and mating occurs right after emergence. After approximately two months of gestation, females lay on average five (range 1–12) soft shelled eggs. Juveniles emerge within one hour after laying and are immediately independent [37]. The lizards used in this study were captured in 2010 from natural populations of the Cévennes mountains (Lozère, France, 44°27' N, 3°44' E, Licence no.2010-189-16 DREAL), marked by toe clipping, and translocated to the Metatron, an infrastructure composed of seminatural caged enclosures located at the Station of Experimental Ecology in Moulis (Ariège, France, 43°01' N, 1°05' E). This unique structure offers 48 interconnected enclosures, each measuring 10 x 10 m, containing natural lizard habitat (dense vegetation, hiding places, and rocks [30,56,71–76], Fig 1). Each enclosure is delimited by tarpaulins buried 30 cm into the ground, preventing escape and terrestrial predation [30], and are fully enclosed with a net preventing avian predation and allowing isolation of each enclosure (Fig 1). Each enclosure acts as a mini ecosystem, with natural vegetation and insect communities and a relatively wide variety of thermal microhabitats (shaded, dense, and diverse vegetation, sun-battered rocks and logs, and ponds, Fig 1). Diversity within these caged habitats is relatively high, with more than 140 vegetal species found within the enclosures for 134 species found in the nearby outside habitat (estimated in May 2014). Considering invertebrate communities, a monitoring allowed to determine more than 123 invertebrate families present in the enclosures against only 106 in the nearby outside habitat (S1 Text, estimated in May 2014). Enclosures can be connected to a 19-m-long one-way corridor with a pitfall trap at the end (Fig 1). This distance corresponds to the minimum dispersal distance of the common lizard [77]. Finally, temperature, illuminance, and hygrometry within each enclosure are monitored every 30 min and can be manipulated through the use of motor-driven shutters and a sprinkler system. Lizards were maintained in the Metatron for two years prior to the experiment in “present climate” conditions (see next section) using similar population densities and structures than in this study.

Between May 2012 and May 2014, we performed two studies manipulating summer climatic conditions and monitoring consequences on lizard populations. We used data from these two years of experiment altogether. The same experimental procedure was used for the two years. From mid-May, at the end of female gestation period, we captured all surviving lizards maintained in the Metatron during multiple successive capture sessions. Each lizard was measured for snout–vent length and total length and weighted. A tail tip was taken for routine genetic sampling. Yearlings (1-year-old lizards) and adult males were kept only for the amount of time necessary to ensure that we had captured all surviving individuals from the enclosures and were released into the Metatron on average one month after capture, whereas females were maintained in the laboratory until parturition. In the laboratory, lizards were kept in 25 x 15.5 x 15 cm individual glass terraria with a 3 cm litter layer, a piece of cardboard and a plastic tube for shelter and a piece of absorbent paper. A light bulb (25 W) and an ultraviolet lamp (Zoomed Reptisun 5.0 UVB 36 W) provided heat for thermoregulation and light 6 h per d (from 9:00 to 12:00 and from 14:00 to 17:00). Lizards were lightly sprayed with water three times a day (in the morning, at mid-day, and in the evening) and offered one cricket (*Acheta domestica*) daily. Between early June and mid-July, females laid eggs in the terraria. Offspring were marked and measured for body length (snout–vent length and total length to the nearest mm) and mass (to the nearest 0.001 g) immediately after birth; their sex was determined by counting ventral scales [78], and a tail tip was taken for genetic sampling. Families were then released into the Metatron.

### Release of the Lizards into the Metatron

Lizards were released into the Metatron controlling for body size and source population. From June to the end of September (2012 and 2013), we applied several climatic treatments to the enclosures. In 2012, we created nine populations from three climatic treatments (three populations in each treatment), while in 2013 we created ten populations from the two extreme climatic treatments (five populations in each treatment). Enclosures were chosen to be the most homogeneous respective to the vegetal cover (F_2,6_ = 0.80, *p* = 0.49 and F_1,8_ = 0.54, *p* = 0.48, respectively for 2012 and 2013), vegetal height (F_2,6_ = 2.26, p = 0.18 and F_1,8_ = 0.04, p = 0.85, respectively for 2012 and 2013), vegetal composition (F_2,6_ = 0.01, *p* = 0.99 and F_1,8_ = 3.16, *p* = 0.11, respectively for 2012 and 2013), and invertebrate prey diversity (F_2,6_ = 0.91, *p* = 0.45 and F_1,8_ = 2.60, *p* = 0.15, respectively for 2012 and 2013, see S1 Text). In 2012, we had a “present climate” (PC) in which automatic shutters were allowed to close when temperature exceeded 28°C, an “intermediate climate” level, in which shutters closed when temperature surpassed 34°C and a “warm climate” (WC) in which shutters were only allowed to close when temperature rose above 38°C. In 2013, we only kept the present and warm climate treatments because the intermediate treatment had similar temperatures and gave similar results to the warm climate treatment. Enclosed habitats are warmer than outside habitats. Closing the shutters both stopped temperature from rising and caused temperatures to drop, evening out temperature peaks. As a result, “present climate” enclosures showed similar summer temperatures to ambient temperatures outside of the Metatron (temperatures in the nearby meteorological station of Saint-Girons Antichan, S2 Text), while “warm climate” enclosures were on average 2°C warmer (e.g., mean daily temperatures between mid-June and mid-September 2012 and 2013, PC: 26.4 ± 0.3°C, WC: 28.3 ± 0.3°C, mean ± SE, F_1,282_ = 23.1, *p*-value < 0.001; maximum daily temperatures: PC: 29.2 ± 0.3°C, WC: 32.1 ± 0.3°C mean ± SE, F_1,282_ = 50.6, *p*-value < 0.001, see S2 Text, S1 Fig, S2 Fig). Our treatments generated significant differences over the summer in temperature and illuminance, but not in hygrometry (S2 Text, S1 Table), while the treatment effects were negligible during the winter and the spring (S2 Table). Such temperature differences are coherent with IPCC climate change projections for southern Europe [29], which predicts a 3°C temperature increase by 2080, with the largest warming during the summer. Indeed, projections from RCP 4.5 scenario (an emission stabilization scenario) in southern Europe predict a temperature increase of between 1.2 and 5.5°C between June and August against −0.2 and 3.0°C between December and February [29]. Thanks to a dense and diverse vegetation, there was a large temporal and spatial variation within enclosures of warm and present climate allowing cooler refuges despite an overall warmer environment (S2 Text). In 2012, we only had three enclosures in the intermediate climate level, and in one of them, a technical problem (important disturbance in the enclosure related to maintenance issues of the Metatron) caused a quasiextinction of a population. Moreover, when we compared summer temperatures between “intermediate” and “warm” climate treatments, we did not find significant differences in mean, maximum, or minimum temperatures (S2 Text). Hence, we decided to exclude the data from the quasiextinct enclosure and merge the data from the two remaining intermediate climate enclosures to the warm climate enclosures for the analyses.

Each year, populations were composed of 11 ± 1 adult females, 6 ± 1 adult males, 9 ± 2 yearlings and 38 ± 4 juveniles. These population densities conform with local densities observed in natural populations [37,79] and in other seminatural experiments on common lizards [68,71,77,80–82]. There was no difference between treatments in juvenile birthdate, in individual snout–vent length, or mass at release (*p* > 0.36 for all).

### Population Monitoring

In mid-July, one-way corridors between enclosures were opened to allow lizard dispersal from enclosures. A pitfall trap at the end of each corridor allowed the capture of dispersing individuals. Dispersing individuals were measured, weighed, and released into another enclosure at random.

In mid-September, we performed three capture–recapture sessions to measure lizard body growth and survival in each enclosure. In these three sessions, we were able to capture 93% of survivors (capture probability estimated by MARK version 6.1 [83]). All surviving lizards were measured for snout–vent length and total length, weighed, and released into their enclosure to hibernate in the Metatron. During these capture sessions in 2012, we caught 12 neonate juveniles born in the enclosures during the summer. A tail tip was taken from these individuals to assess maternity and paternity.

Finally, the following spring, we recaptured all surviving lizards from each enclosure during multiple capture sessions (>10) without release and brought them into the laboratory. All surviving lizards were measured and weighed again and kept in similar conditions as described above until female parturition, allowing assessment of female reproductive success.

### Genetic Data and Maternities

Genomic DNA of females and neonate juveniles was extracted from tail tips using the QIAquick 96 Purification Kit (QIAGEN) according to the manufacturer’s instructions after a digestion of tissue samples with proteinase K. Individuals were genotyped using eight microsatellite markers [78]. We checked for perfect match between juveniles and their assessed maternities (no mismatch between female and juvenile) using CERVUS software, v.3.0 (see [78] for details on methodology). The 12 neonate juveniles born in the Metatron during the summer 2012 were assigned to five females. These females had already produced a first clutch during their stay in the laboratory in June, and neonates found were born from a second clutch during the summer.

### Statistical Analyses

We modeled the effect of climatic treatment on individual dispersal probability, survival probability, body growth (difference between snout–vent length at release at the beginning of the experiment and snout–vent length at capture), body condition (residuals from a linear model of body mass by body length), and finally on female probability of gravidity (probability that a female will lay eggs), clutch size (number of viable offspring laid by a gravid female), and laying date (treated as a continuous variable). We analyzed juvenile data separately from adult and yearling data, since this allowed us to include a family effect in the analysis concerning juveniles, as siblings cannot be considered as independent. We first analyzed dispersal propensity, then we excluded dispersing individuals from the latter analyses, as dispersing individuals could not be assigned to a unique temperature treatment for the whole summer period. For survival probability, body growth rate, and body condition, we analyzed effects of climatic treatments over a year. However, we also provide in S3 Table the effects of treatment by the end of summer in order to better understand paths of effects.

To estimate the effect of temperature treatment on juvenile, yearling, and adult demography, we performed generalized mixed models and linear mixed models with lmer procedure [84] in R, version 3.1.1 [85]. Dispersal, survival, and probability of gravidity were modeled using a generalized mixed model with a binomial distribution and a logit link. Body growth, body condition, and laying date were modeled as linear variables. Finally, clutch size was modeled using a Poisson distribution, except for clutch size in September 2012 where we used a zero-inflated Poisson GLM because of the low number of neonate juveniles recovered in September 2012. Models included temperature treatment as a categorical variable and several covariates plus random intercepts. For juveniles, we included birthdate modeled as a continuous covariate, and for adult and yearlings, we included age modeled as a two-level factorial variable (yearling or adult) and sex. Finally, mixed modeling allowed adding random intercepts to the models: 1) a family effect in juvenile analyses, as juveniles from a family are not independent, 2) enclosure identity to account for variation due to potential differences among enclosures, and 3) the year of experiment to account for the block design. Following Zuur et al. [86], we fitted full models with all fixed variables and every combination of random intercepts with a restricted maximum likelihood approach. We compared models using the respective AIC and chose the best structure of the random component for each dependent variable. We compared a full model with temperature treatment, necessary covariates, and random intercepts to a model including only the covariates and random intercepts through their ΔAIC. We then performed likelihood ratio tests to evaluate the impact of the temperature treatment. We provided estimates and standard errors of the effect of each fixed variable. We further calculated both the marginal (effect of the fixed variables) and the conditional (effect of the fixed and random variables) R², as well as the PCV for each random variable following Nakagawa and Schielzeth [87].

Adults survived less in warmer conditions; hence we tested for the impact on adult density in September on juvenile survival and body growth. Similarly, juveniles grew more in warmer conditions, thus we also tested for the impact of their body growth in September on winter survival. Finally, we checked that shifts in invertebrate communities due to warming climates could not explain the lower adult survival in warm climate enclosures. There were no differences between warm and present enclosures in the number of insect families (F_1,17_ = 0.37, *p* = 0.55), or in the density of insects (F_1,17_ = 0.17, *p* = 0.69) or arachnids (F_1,17_ = 0.02, *p* = 0.89) after one year; therefore, it was unlikely that differences in prey availability could lead to differences in survival. Nevertheless, we tested the impact of insect density the following year on adult survival and on juvenile survival and body growth.

## Supporting Information

S1 Data Raw data spreadsheet for juveniles. (XLSX)

S2 Data Raw data spreadsheet for adults and yearlings. (XLSX)

S1 Fig Maximum daily temperatures (°C ± SE) between June and September in the two temperature treatments. (PNG)

S2 Fig Mean daily temperatures during lizard activity period (°C ± SE) between June and September in the two temperature treatments. (PNG)

S3 Fig Three age class life cycle graphs representing lizard population dynamics in our system. s_j_: juvenile survival, s_y_: yearling survival, s_a_: adult survival, p_y_: yearling probability of gravidity, p_a_: adult probability of gravidity, f_y_: yearling fecundity, f_a_: adult fecundity, σ: primary sex ratio.
(PDF)

S1 Table Impact of climatic treatment on our principal climatic variables during the summer (June–September). ANOVAs compare values between present climate and warm climate (mix of warm and intermediate climate treatments). Summaries of the mean values ± SE of the parameters are given for each treatment.
(DOCX)

S2 Table Impact of climatic treatment on our principal climatic variables during the winter and spring (January–May). ANOVAs compare values between present climate and warm climate (mix of warm and intermediate climate treatments). Summaries of the mean values ± SE of the parameters are given for each treatment.
(DOCX)

S3 Table Effect of temperature treatment on juvenile, yearling, and adult dispersal and on summer survival, body growth, and body condition. (DOCX)

S4 Table Values for each demographic parameter estimated from the outputs of the mixed models investigating the impact of climatic treatment. (DOCX)

S5 Table Sensitivity and elasticity of λ to the values of each demographic parameter. (DOCX)

S6 Table Position and averaged maximum daily temperatures observed for each common lizard populations, associated “risk levels” (from A: imminent risk to F: low risk), and references for the global positioning system coordinates, plus extinction status from Sinervo et al. 2010 survey populations (0 = recently extinct and committed to extinction populations; 1 = populations maintaining themselves). (DOCX)

S7 Table Data underlying Fig 2. (DOCX)

S1 Text Invertebrate community sampling. (DOCX)

S2 Text Temperature treatments. (DOCX)

S3 Text Population dynamics modeling. (DOCX)

S4 Text Projections for European populations of common lizards. (DOCX)

## Abbreviations

IPCC: Intergovernmental Panel on Climate Change
RCP: Representative Concentration Pathway
PCV: proportion change in variance
SE: Standard Error
