# Supplementary figures and images for: Live Fast, Die Young: Experimental Evidence of Population Extinction Risk due to Climate Change
Source: PLoS Biol. 2015 Oct 26;13(10):e1002281. doi: 10.1371/journal.pbio.1002281 (PMC4621050; doi:10.1371/journal.pbio.1002281)

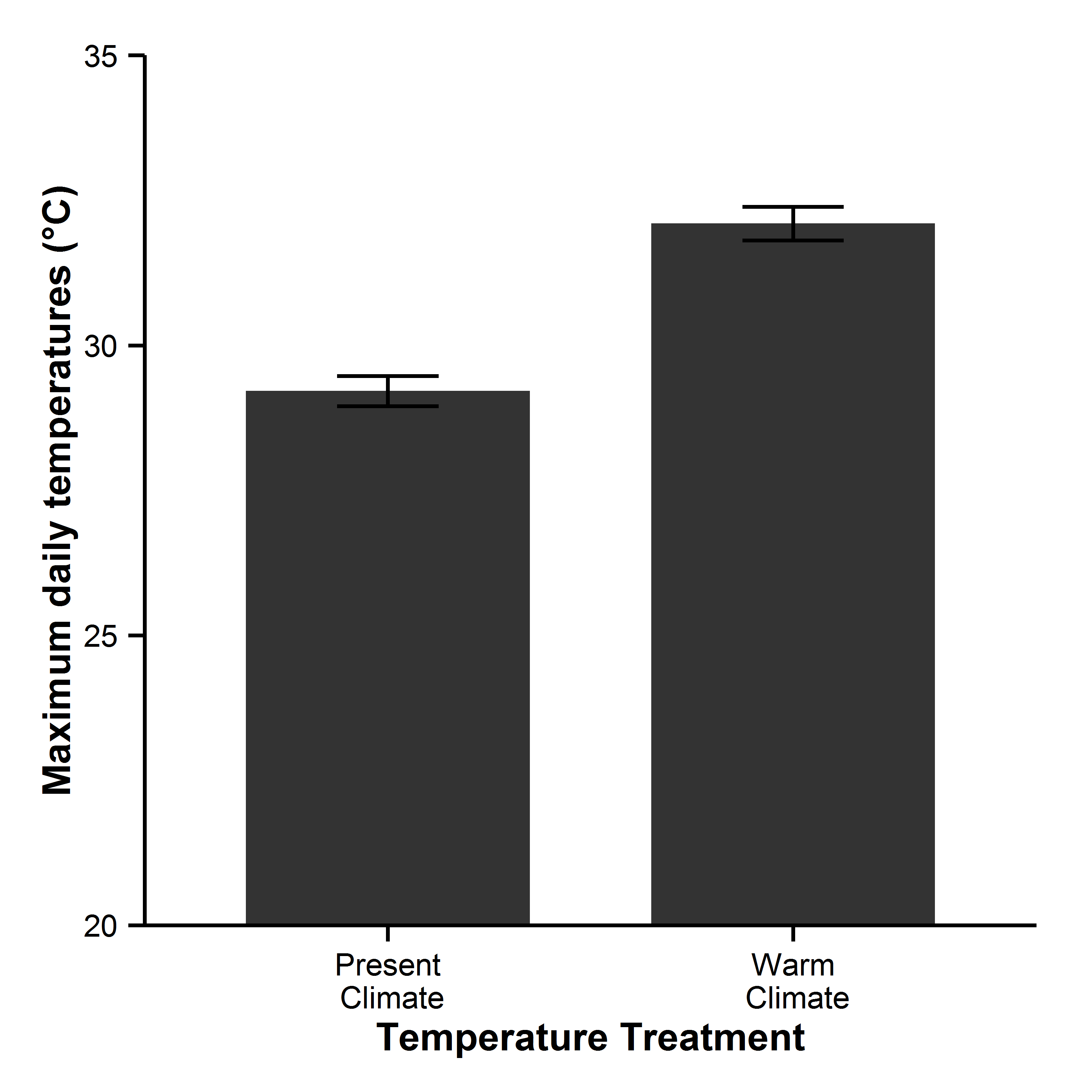

Supplement: S1 Fig — (PNG) [file pbio.1002281.s003.png]

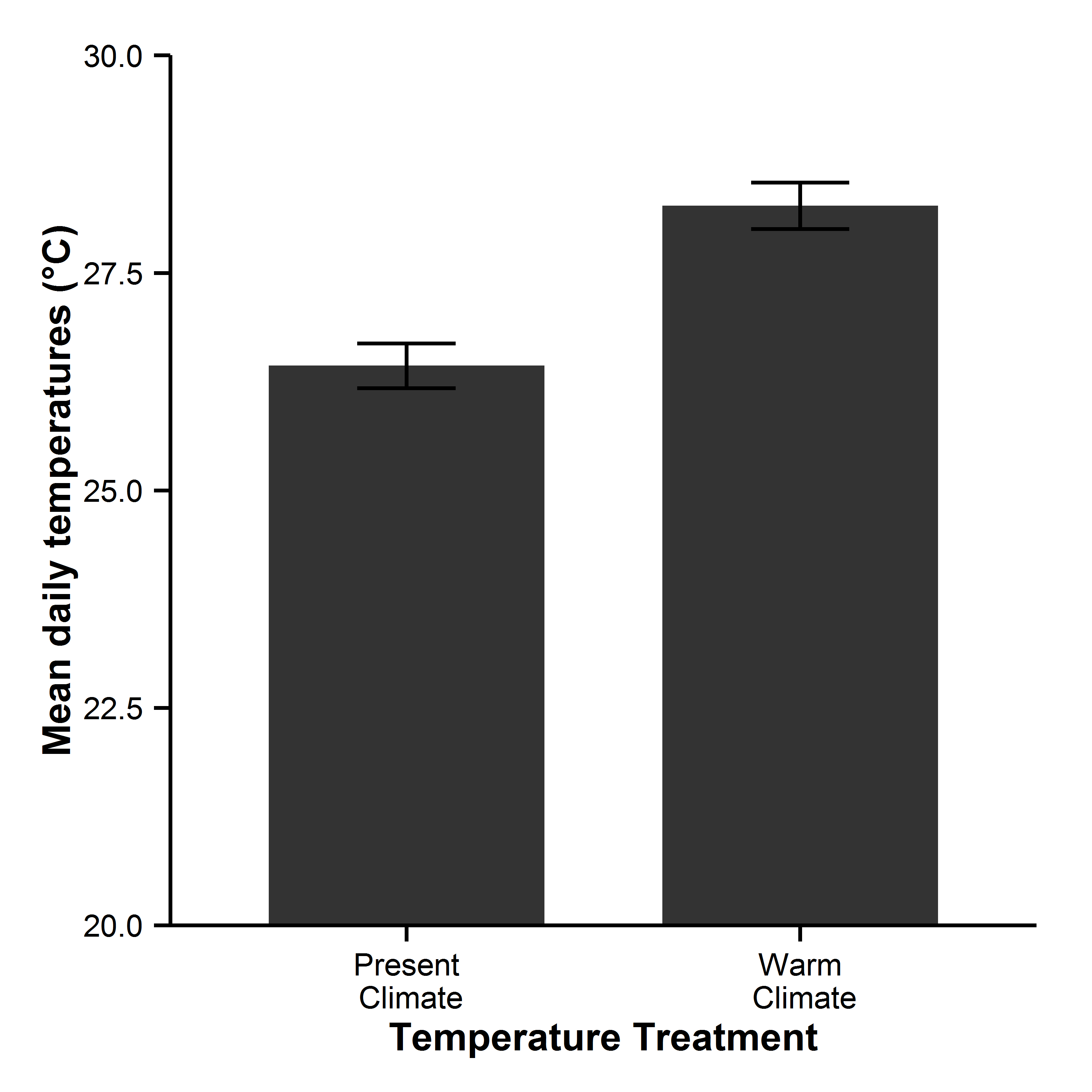

Supplement: S2 Fig — (PNG) [file pbio.1002281.s004.png]

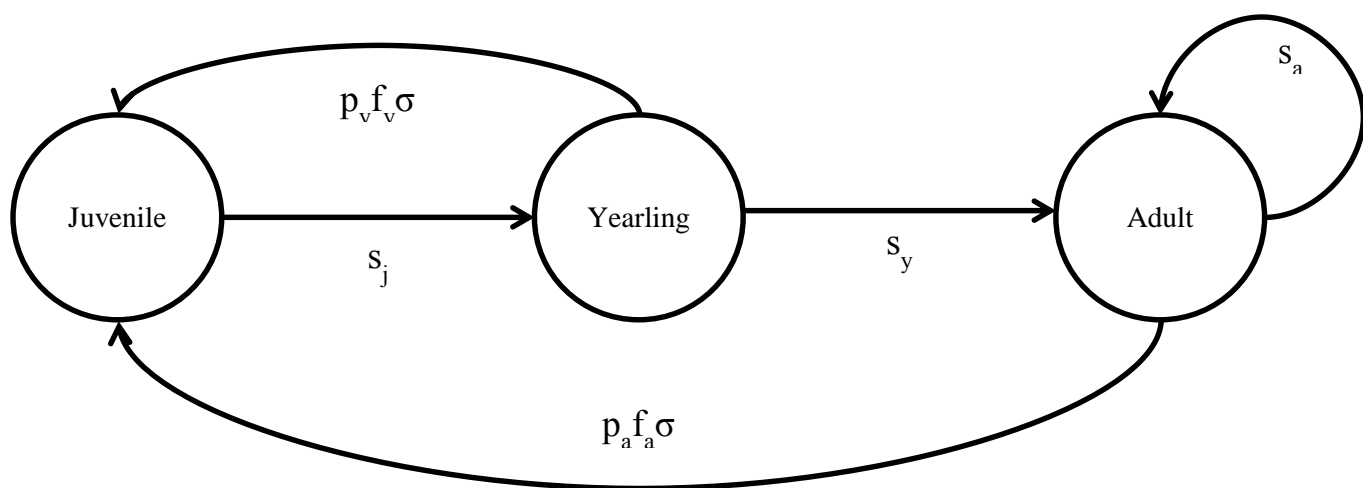

Supplement: S3 Fig — sj: juvenile survival, sy: yearling survival, sa: adult survival, py: yearling probability of gravidity, pa: adult probability of gravidity, fy: yearling fecundity, fa: adult fecundity, σ: primary sex ratio. (PDF) [file pbio.1002281.s005.pdf]
