# Supplementary material for: Live Fast, Die Young: Experimental Evidence of Population Extinction Risk due to Climate Change
Source: PLoS Biol. 2015 Oct 26;13(10):e1002281. doi: 10.1371/journal.pbio.1002281 (PMC4621050; doi:10.1371/journal.pbio.1002281)
Supplement: S1 Table — ANOVAs compare values between present climate and warm climate (mix of warm and intermediate climate treatments). Summaries of the mean values ± SE of the parameters are given for each treatment. (DOCX) [file pbio.1002281.s006.docx]

| **Variable** | **Present Climate** | **Warm Climate** | **Df** | **F value** | **p-value** |
| --- | --- | --- | --- | --- | --- |
|  |  |  |  |  |  |
| **Temperature** |  |  |  |  |  |
| Maximum daily temperature | 29.21 ± 0.26 | 32.10 ± 0.29 | 1,282 | 50.6 | <0.001 |
| Mean daily temperature | 26.43 ± 0.26 | 28.27 ± 0.27 | 1,282 | 23.1 | <0.001 |
| Minimum daily temperature | 21.93 ± 0.28 | 22.26 ± 0.24 | 1,282 | 0.83 | 0.361 |
| Nb of hours over CTMax | 0.00 ± 0.00 | 0.06 ± 0.02 | 1,282 | 6.52 | 0.011 |
| Mean nightly temperature | 13.92 ± 0.19 | 13.58 ± 0.17 | 1,282 | 1.6 | 0.207 |
| SD daily temperature | 2.21 ± 0.05 | 3.02 ± 0.06 | 1,282 | 102 | <0.001 |
| **Illuminance** |  |  |  |  |  |
| Mean daily illuminance | 30157 ± 1200 | 47352 ± 788 | 1,281 | 249 | <0.001 |
| **Hygrometry** |  |  |  |  |  |
| Mean daily hygrometry | 58.50 ± 0.68 | 53.29 ± 0.68 | 1,274 | 28.2 | <0.001 |
