# Supplementary material for: Live Fast, Die Young: Experimental Evidence of Population Extinction Risk due to Climate Change
Source: PLoS Biol. 2015 Oct 26;13(10):e1002281. doi: 10.1371/journal.pbio.1002281 (PMC4621050; doi:10.1371/journal.pbio.1002281)
Supplement: S2 Table — ANOVAs compare values between present climate and warm climate (mix of warm and intermediate climate treatments). Summaries of the mean values ± SE of the parameters are given for each treatment. (DOCX) [file pbio.1002281.s007.docx]

| **Variable** | **Present Climate** | **Warm Climate** | **Df** | **F value** | **p-value** |
| --- | --- | --- | --- | --- | --- |
|  |  |  |  |  |  |
| **Temperature** |  |  |  |  |  |
| Maximum daily temperature | 19.15 ± 0.46 | 20.38 ± 0.47 | 1,293 | 3.39 | 0.066 |
| Mean daily temperature | 15.47 ± 0.44 | 15.93 ± 0.43 | 1,293 | 0.55 | 0.46 |
| Minimum daily temperature | 10.34 ± 0.40 | 10.02 ± 0.39 | 1,293 | 0.31 | 0.576 |
| Mean nightly temperature | 5.21 ± 0.27 | 4.97 ± 0.26 | 1,293 | 0.41 | 0.524 |
| **Illuminance** |  |  |  |  |  |
| Mean daily illuminance | 11612 ± 604 | 13998 ± 669 | 1,302 | 6.56 | 0.011 |
| **Hygrometry** |  |  |  |  |  |
| Mean daily hygrometry | 65.57 ± 1.18 | 63.57 ± 1.20 | 1,299 | 1.4 | 0.238 |
