# Supplementary material for: Live Fast, Die Young: Experimental Evidence of Population Extinction Risk due to Climate Change
Source: PLoS Biol. 2015 Oct 26;13(10):e1002281. doi: 10.1371/journal.pbio.1002281 (PMC4621050; doi:10.1371/journal.pbio.1002281)
Supplement: S3 Table — (DOCX) [file pbio.1002281.s008.docx]

|  | Best Model | Likelihood ratio test (df = 1) | |  | Effect of the temperature treatment | |
| --- | --- | --- | --- | --- | --- | --- |
|  |  | χ² | p |  | Estimate | SE |
| **Adults and yearlings** |  |  |  |  |  |  |
| Dispersal probability | age+sex+(1\|Enclosure)+(1\|Year) | 0.04 | 0.841 |  |  |  |
| Summer survival | **Temperature**+age+sex+(1\|Enclosure) | 4.56 | 0.033 * |  | -0.48 | 0.24 |
| Summer body growth | age+sex+(1\|Year) | 0.04 | 0.840 |  |  |  |
| Summer body condition | age+sex+(1\|Enclosure) | 0.04 | 0.843 |  |  |  |
| **Juveniles** |  |  |  |  |  |  |
| Dispersal probability | date of birth+(1\|Enclosure) | 0.19 | 0.659 |  |  |  |
| Summer survival | Temperature+date of birth+(1\|Enclosure)+(1\|Family) | 3.58 | 0.058 . |  | -0.43 | 0.23 |
| Summer body growth | **Temperature**+date of birth+(1\|Enclosure)+(1\|Family)+(1\|Year) | 14.99 | <0.001 *** |  | 2.90 | 0.72 |
| Summer body condition | date of birth+(1\|Enclosure)+(1\|Family)+(1\|Year) | 1.80 | 0.180 |  |  |  |

NOTE: Statistics of Likelihood Ratio test compare two models, one with temperature treatment and one simpler model without temperature treatment. GLMM with logit links are used for binomial factors such as dispersal and survival, other variables are modeled with linear mixed models. When the best model includes temperature treatment, we provide estimate and standard error of the temperature treatment.
