# Supplementary material for: Live Fast, Die Young: Experimental Evidence of Population Extinction Risk due to Climate Change
Source: PLoS Biol. 2015 Oct 26;13(10):e1002281. doi: 10.1371/journal.pbio.1002281 (PMC4621050; doi:10.1371/journal.pbio.1002281)
Supplement: S4 Table — (DOCX) [file pbio.1002281.s009.docx]

| Demographic parameter |  | Present climate treatment | |  | Warm climate treatment | |
| --- | --- | --- | --- | --- | --- | --- |
|  |  | Estimate | SE |  | Estimate | SE |
| Survival |  |  |  |  |  |  |
| s_j_ |  | 0.274 | 0.007 |  | 0.176 | 0.005 |
| s_y_ |  | 0.458 | 0.012 |  | 0.347 | 0.014 |
| s_a_ |  | 0.561 | 0.009 |  | 0.454 | 0.011 |
| Probability of gravidity |  |  |  |  |  |  |
| p_y_ |  | 0.371 | 0.016 |  | 0.600 | 0.020 |
| p_a_ |  | 0.873 | 0.012 |  | 0.984 | 0.001 |
| Fecundity |  |  |  |  |  |  |
| f_y_ |  | 4.221 | 0.136 |  | 3.459 | 0.109 |
| f_a_ * |  | 5.884 | 0.032 |  | 5.581/5.803 | 0.035 |

* for warm climate treatment, adult female fecundity was considered first without then with second clutches
