# Supplementary material for: Live Fast, Die Young: Experimental Evidence of Population Extinction Risk due to Climate Change
Source: PLoS Biol. 2015 Oct 26;13(10):e1002281. doi: 10.1371/journal.pbio.1002281 (PMC4621050; doi:10.1371/journal.pbio.1002281)
Supplement: S5 Table — (DOCX) [file pbio.1002281.s010.docx]

| Demographic parameter |  | Present climate treatment | |  | Warm climate treatment | |
| --- | --- | --- | --- | --- | --- | --- |
|  |  | Sensitivity | Elasticity |  | Sensitivity | Elasticity |
| Survival |  |  |  |  |  |  |
| s_j_ |  | 1.268 | 0.354 |  | 1.461 | 0.348 |
| s_y_ |  | 0.593 | 0.277 |  | 0.558 | 0.259 |
| s_a_ |  | 0.646 | 0.370 |  | 0.658 | 0.400 |
| Probability of gravidity |  |  |  |  |  |  |
| p_y_ |  | 0.204 | 0.078 |  | 0.103 | 0.083 |
| p_a_ |  | 0.311 | 0.277 |  | 0.197 | 0.259 |
| Fecundity |  |  |  |  |  |  |
| f_y_ |  | 0.018 | 0.077 |  | 0.018 | 0.083 |
| f_a_ |  | 0.046 | 0.277 |  | 0.035 | 0.259 |
