# Supplementary material for: Live Fast, Die Young: Experimental Evidence of Population Extinction Risk due to Climate Change
Source: PLoS Biol. 2015 Oct 26;13(10):e1002281. doi: 10.1371/journal.pbio.1002281 (PMC4621050; doi:10.1371/journal.pbio.1002281)
Supplement: S6 Table — (DOCX) [file pbio.1002281.s011.docx]

| **Locality** | **Country** | **Latitude** | **Longitude** | **Average Maximum Temperature** | **Risk profile** | **Extinction Status** | **References** |
| --- | --- | --- | --- | --- | --- | --- | --- |
| Chiareggio | ITA | 46.317 | 9.788261 | 12.13 | F |  | [1] |
| Vallorcine | FRA | 45.9967 | 6.870952 | 17.73 | F |  | [1] |
| Moncenisio | ITA | 45.2033 | 6.984325 | 20.22 | F |  | [1] |
| Hausalm | AUT | 46.7259 | 10.85778 | 16.79 | F |  | [1] |
| Passo Pordoi | ITA | 46.4873 | 11.81166 | 17.12 | F |  | [1] |
| Emberger Alm | AUT | 46.7745 | 13.15578 | 17.01 | F |  | [1] |
| Serfaus | AUT | 47.03 | 10.55 | 17.78 | F |  | [2] |
| Turracher Höhe | AUT | 46.9086 | 13.87861 | 18.40 | F | 1 | [2] |
| Berne Foralps | CHE | 46.58 | 7.66 | 20.04 | F |  | [3] |
| Passo Giau | ITA | 46.486 | 12.05002 | 19.72 | F |  | [1] |
| Sara Mt. | SCG | 41.83 | 20.67 | 23.30 | F |  | [3] |
| Rila-Belli Iskar | BGR | 42.1056 | 23.51942 | 22.49 | F |  | [1] |
| Forni Avoltri | ITA | 46.5855 | 12.77738 | 20.03 | F | 1 | [2] |
| Retezat | ROU | 45.3467 | 22.82413 | 22.73 | F |  | [1] |
| Pourtalet | FRA | 42.7953 | -0.40384 | 22.24 | F | 1 | [2,4] |
| Brousset | FRA | 42.8513 | -0.38935 | 22.24 | F | 1 | [2,4] |
| Lac d’Artouste | FRA | 42.8634 | -0.33594 | 22.24 | F | 1 | [2] |
| Plateau du Soussouéou | FRA | 42.9014 | -0.36673 | 22.24 | F | 1 | [2] |
| Col d'Aubisque | FRA | 42.9725 | -0.34547 | 22.24 | F | 1 | [2] |
| Oropa | ITA | 45.6258 | 7.9838 | 21.70 | F | 1 | [2] |
| Chalet à Roch | CHE | 46.5728 | 6.188024 | 22.46 | F |  | [1] |
| Brassus | CHE | 46.5822 | 6.211889 | 22.46 | F |  | [1] |
| Lac Montcineyre | FRA | 45.46 | 2.9 | 22.27 | F |  | [3] |
| Šunava | SK | 49.01 | 20.11 | 20.19 | F |  | [3] |
| Lac Pavin | FRA | 45.5 | 2.91 | 22.41 | F |  | [3] |
| Col de la Moréno | FRA | 45.73 | 2.93 | 22.41 | F |  | [3] |
| Montselgues | FRA | 44.5113 | 3.99163 | 23.53 | F | 1 | [2] |
| Bjelasica | SCG | 42.8537 | 19.70792 | 25.01 | F |  | [1] |
| Le Chalet du Mont Lozère | FRA | 44.48 | 3.72 | 23.64 | F |  | [3] |
| Podkoren-Zelenci | SI | 46.4926 | 13.73773 | 22.80 | F | 1 | [2] |
| Carinthia | AUT | 46.58 | 13.13 | 22.36 | F |  | [3] |
| Semmering | AUT | 47.6406 | 15.82242 | 21.69 | F |  | [1] |
| Breitenstein | AUT | 47.658 | 15.81973 | 21.69 | F |  | [1] |
| Vevey | CHE | 46.4736 | 6.85203 | 24.10 | F |  | [1] |
| Frasne | FRA | 46.8227 | 6.15818 | 22.96 | F | 1 | [2] |
| Charbonnières | CHE | 46.6665 | 6.315135 | 23.68 | F |  | [1] |
| Apuseni | ROU | 46.6175 | 22.76439 | 24.10 | F |  | [1] |
| Cirque de Lescun | FRA | 42.9012 | -0.64748 | 24.32 | F | 1 | [2] |
| Sureanu Mts. | ROU | 45.6 | 23.3 | 25.48 | F |  | [3] |
| St Rivoal | FRA | 48.3498 | -3.99824 | 19.59 | F |  | [1] |
| Rodnei | ROU | 47.5684 | 24.70314 | 23.90 | F |  | [1] |
| Chatel Saint Denis | CHE | 46.518 | 6.981699 | 23.90 | F |  | [1] |
| Mas de la Barque | FRA | 44.38 | 3.82 | 25.01 | F |  | [3] |
| Plateau de la Croix de l'Ermite | FRA | 44.38 | 3.89 | 25.01 | F |  | [3] |
| Pirin | BGR | 41.7638 | 23.39937 | 26.47 | F |  | [1] |
| Foret de Pinet | FRA | 42.8729 | 1.98038 | 25.01 | F | 1 | [2] |
| Cernisko | SI | 45.7397 | 14.39072 | 25.75 | F | 0 | [2] |
| Mountain Vlašic | BH | 44.28 | 17.63 | 27.78 | D |  | [3] |
| La Traverse | FRA | 42.9343 | 1.11834 | 25.01 | F | 0 | [2] |
| Col de Port | FRA | 42.9031 | 1.44571 | 25.18 | F | 1 | [2] |
| Kiruna | SWE | 67.8595 | 20.22509 | 15.03 | F |  | [1] |
| Vysoká Hole | CZK | 50.033 | 17.2 | 21.92 | F |  | [2] |
| Žiar | SK | 49.13 | 19.66 | 23.16 | F |  | [3] |
| Oulanka | FIN | 66.37 | 29.32 | 18.04 | F |  | [3] |
| Caroux | FRA | 43.6025 | 2.98223 | 26.18 | F | 1 | [2] |
| Tourbiere de Planisie | FRA | 43.6748 | 2.99807 | 26.18 | F | 0 | [2] |
| Tour du Vialat | FRA | 44.3382 | 2.87534 | 26.33 | F | 1 | [2] |
| Priddy | GB | 51.25 | -2.66 | 19.81 | F |  | [3] |
| Bristol | GB | 51.4624 | -2.58934 | 19.81 | F |  | [1] |
| Balkan-Petrohan | BGR | 43.1227 | 23.12518 | 28.19 | D |  | [1] |
| Chambery | FRA | 45.5343 | 5.924378 | 27.50 | E |  | [1] |
| Stara Mt. | SCG | 43.37 | 22.75 | 28.53 | D |  | [3] |
| Valle San Nicolo | ITA | 45.6079 | 8.141521 | 23.70 | F |  | [1] |
| Them | DEN | 56.0874 | 9.54289 | 19.87 | F |  | [1] |
| Rila-Govedarci | BGR | 42.2607 | 23.47419 | 28.83 | C |  | [1] |
| Tourbiere de La Rhune | FRA | 43.3157 | -1.6273 | 25.95 | F | 1 | [2] |
| Lac de Barandon | FRA | 44.4423 | 2.38167 | 27.12 | E | 1 | [2] |
| Chalet | FRA | 44.4498 | 2.25214 | 27.12 | E | 1 | [2] |
| Runsten | SWE | 56.7163 | 16.69362 | 21.21 | F |  | [1] |
| Markitta | SWE | 67.16 | 21.5 | 17.49 | F |  | [3] |
| Issarbes | FRA | 43.031 | -0.797 | 26.93 | E | 1 | [2,4] |
| England | GB | 51.333 | -1.417 | 20.65 | F |  | [2] |
| Plateau de Ger | FRA | 43.1736 | -0.06851 | 26.78 | E | 0 | [2,4] |
| Kalmthoutse Heide | BEL | 51.41 | 4.41 | 23.21 | F |  | [3] |
| Oberdrees | DEU | 50.63 | 6.91 | 23.09 | F |  | [3] |
| Paimpont | FRA | 48 | -2.17 | 22.81 | F |  | [2,3] |
| Plateau d’Aran | FRA | 43.0468 | -0.52235 | 26.98 | E | 1 | [2] |
| Col de Marie-Blanque | FRA | 43.0708 | -0.50787 | 26.98 | E | 1 | [2,4] |
| Ustrzyki Gorne | POL | 49.4279 | 22.62115 | 24.64 | F |  | [1] |
| Winchester | GB | 51.0626 | -1.30963 | 20.82 | F |  | [1] |
| Umea | SWE | 63.83 | 20.26119 | 19.17 | F |  | [1] |
| Radun | CZK | 49.883 | 17.2 | 24.02 | F |  | [2] |
| Moura de Montrol | FRA | 43.513 | -1.29636 | 26.55 | F | 0 | [2,4] |
| Szklarska Poreba | POL | 50.8183 | 15.50587 | 22.83 | F |  | [1] |
| Vitocha | BGR | 42.5669 | 23.28329 | 29.82 | B |  | [1] |
| Sandsjöbacka | SWE | 57.53 | 12.03 | 19.71 | F |  | [3] |
| Öjersjö | SWE | 57.7 | 12.13 | 19.71 | F |  | [3] |
| Rambouillet | FRA | 48.6728 | 1.811873 | 24.43 | F |  | [1] |
| Helsinki | FIN | 60.17 | 24.88 | 20.70 | F |  | [3] |
| Valdeasa | ROU | 46.76 | 22.79 | 28.03 | D |  | [1] |
| Col des Palomieres | FRA | 43.0569 | 0.19296 | 27.35 | E | 1 | [2,4] |
| Jyväskylä | FIN | 62.18 | 25.33 | 20.17 | F |  | [3] |
| Jyväskylä | FIN | 62.1 | 25.45 | 20.17 | F |  | [3] |
| Plateau du Benou | FRA | 43.0632 | -0.45656 | 27.35 | E | 1 | [2] |
| Louvie-Pedestarres | FRA | 43.093 | -0.38039 | 27.35 | E | 1 | [2] |
| Tourbiere de Buzy | FRA | 43.1506 | -0.44847 | 27.35 | E | 0 | [2] |
| Petäjävesi | FIN | 62.17 | 25.7 | 20.30 | F |  | [3] |
| Overasseltse-Haterste Vennen | NL | 51.7863 | 5.800672 | 23.26 | F |  | [1] |
| Ostravice | CZK | 49.567 | 18.417 | 25.29 | F |  | [2] |
| Kolonica | SK | 48.9544 | 22.25326 | 26.11 | F |  | [1] |
| De Hamert | NL | 51.53 | 6.17 | 23.79 | F |  | [3] |
| Troubiere de Uzein | FRA | 43.3867 | -0.33294 | 27.20 | E | 0 | [2] |
| Cernovits | UKR | 48.2926 | 25.93355 | 28.19 | D |  | [1] |
| Matsalu | EST | 58.7378 | 23.68302 | 21.80 | F |  | [1] |
| Puy Mary - Crater | FRA | 45.1122 | 1.31382 | 27.98 | D | 1 | [2] |
| Bonnevaux | FRA | 44.3707 | 4.030269 | 29.03 | C |  | [1] |
| Eremitu | ROU | 46.6589 | 24.92491 | 29.92 | B |  | [1] |
| Krutyn | POL | 53.6888 | 21.43095 | 23.46 | F |  | [1] |
| Trebon | CZK | 49.0062 | 14.78995 | 25.36 | F |  | [1] |
| Kopaonik | SCG | 43.7502 | 20.7814 | 31.39 | B |  | [1] |
| Grodno | BLR | 53.6706 | 23.83338 | 23.77 | F |  | [1] |
| Leipzig | DEU | 51.36 | 12.23 | 24.76 | F |  | [3] |
| Slunecná | CZK | 50.833 | 17.467 | 26.27 | F |  | [2] |
| Wiener am See | AUT | 47.8949 | 16.3166 | 27.76 | D |  | [1] |
| Moosbrunn | AUT | 48.0199 | 16.45998 | 27.95 | D |  | [1] |
| Odolanów | POL | 51.57 | 17.66 | 25.29 | F |  | [3] |
| Kiev | UKR | 50.4368 | 30.49287 | 27.61 | D |  | [1] |
| Palude di Moretto | ITA | 45.8975 | 13.16472 | 31.01 | B | 0 | [2] |
| Marghita | ROU | 47.3536 | 22.33631 | 30.56 | B |  | [1] |
| Botany | SK | 48.4466 | 22.09438 | 29.33 | C |  | [1] |
| Felsobesnyo | HUN | 47.2337 | 19.26977 | 30.93 | B | 0 | [2] |
| Markotabödöge-Hansági | HUN | 47.6881 | 17.35133 | 29.33 | C | 0 | [2] |
| Mand-Fulesd | HUN | 48.0021 | 22.64261 | 29.81 | B | 0 | [2] |
| Féhérgyarmat | HUN | 48.0108 | 22.50961 | 29.81 | B | 0 | [2] |
| Tarpa | HUN | 48.1264 | 22.53188 | 29.81 | B | 0 | [2] |
| Bátorliget | HUN | 47.7692 | 22.26628 | 30.16 | B | 1 | [2] |
| Fabianhaza | HUN | 47.8439 | 22.33018 | 30.16 | B |  | [1] |
| Ócsa u Budapest | HUN | 47.2704 | 19.22784 | 30.66 | B | 0 | [2] |
| Busatello | ITA | 45.101 | 11.093 | 33.95 | A | 1 | [2] |
| Bulgarian Mts. | BGR | 41 | 23 | 35.20 | A |  | [3] |
| Asketunnan, Hållsundsudde | SWE | 57.36 | 11.96 |  |  |  | [3] |
| Anglesey | GB | 53.2786 | -4.4304 |  |  |  | [1] |

**References**

1. Surget-Groba Y, Heulin B, Guillaume C-P, Puky M, Semenov D, Orlova V, et al. Multiple origins of viviparity, or reversal from viviparity to oviparity? The European common lizard (Zootoca vivipara, Lacertidae) and the evolution of parity. Biol J Linn Soc. 2006;87: 1–11. doi:10.1111/j.1095-8312.2006.00552.x

2. Sinervo B, Méndez-de-la-Cruz F, Miles DB, Heulin B, Bastiaans E, Villagrán-Santa Cruz M, et al. Erosion of lizard diversity by climate change and altered thermal niches. Science. 2010;328: 1354–1354. doi:10.1126/science.1184695

3. Horváthová T, Cooney CR, Fitze PS, Oksanen TA, Jelić D, Ghira I, et al. Length of activity season drives geographic variation in body size of a widely distributed lizard. Ecol Evol. 2013;3: 2424–2442. doi:10.1002/ece3.613

4. Heulin B, Surget-Groba Y, Sinervo B, Miles D, Guiller A. Dynamics of haplogroup frequencies and survival rates in a contact zone of two mtDNA lineages of the lizard Lacerta vivipara. Ecography. 2011;34: 436–447. doi:10.1111/j.1600-0587.2010.06540.x
