# Supplementary material for: Live Fast, Die Young: Experimental Evidence of Population Extinction Risk due to Climate Change
Source: PLoS Biol. 2015 Oct 26;13(10):e1002281. doi: 10.1371/journal.pbio.1002281 (PMC4621050; doi:10.1371/journal.pbio.1002281)
Supplement: S7 Table — (DOCX) [file pbio.1002281.s012.docx]

|  | Present climate | | | Warm climate | | |
| --- | --- | --- | --- | --- | --- | --- |
|  | N | Mean value | SE | N | Mean value | SE |
| Juvenile annual growth rate (mm) | 74 | 29.7 | 0.5 | 60 | 32.8 | 0.5 |
| Female juveniles gravidity probability | 35 | 0.37 | 0.08 | 25 | 0.60 | 0.10 |
| Adult and yearling annual survival | 189 | 0.52 | 0.04 | 242 | 0.42 | 0.03 |
| Adult summer clutch size | 0 | NA | NA | 5 | 2.4 | 0.7 |
