# Supplementary material for: Live Fast, Die Young: Experimental Evidence of Population Extinction Risk due to Climate Change
Source: PLoS Biol. 2015 Oct 26;13(10):e1002281. doi: 10.1371/journal.pbio.1002281 (PMC4621050; doi:10.1371/journal.pbio.1002281)
Supplement: S1 Text — (DOCX) [file pbio.1002281.s013.docx]

In May 2012, 2013 and 2014, we surveyed the arthropod community in each enclosure of the Metatron. We set up two pitfall traps (Ø 8 x 12 cm glass jars filled with 40 % ethanol with a drop of detergent to break water tension surface) buried in the soil of each enclosure. The number of traps within enclosures corresponded to densities of traps found in the literature [1]. The pitfall traps were placed at least 1.5 m from the border of the enclosures to prevent edge effects, in vegetation areas representative of the vegetation within the enclosures, and separated by at least 5 m. Jars were maintained closed inside the enclosures over years so as to prevent biases dues to changes in sampling locations. In June of each year, during a sunny period, we opened the pit-fall traps and let them for 5 days to trap crawling arthropods within each enclosure. Pitfalls were then recovered and arthropods were sorted out and preserved in 70 % ethanol for later identification.

In parallel we performed two net-sampling sessions with a 25 cm diameter sweep net to recover arthropods in higher vegetation stratums and flying insects. Sampling sessions were done in sunny days. Sampling in every enclosure was done by the same experimenter, who performed a U sampling within the enclosure. Recovered arthropods were preserved in 70 % ethanol.

In the laboratory, arthropods were identified to the family level. Data from paired pitfall traps in each enclosure were pooled.

**References**

1. House GJ, Stinner BR. Arthropods in no-tillage soybean agroecosystems: Community composition and ecosystem interactions. Environ Manage. 1983;7: 23–28. doi:10.1007/BF01867037
