# Supplementary material for: Live Fast, Die Young: Experimental Evidence of Population Extinction Risk due to Climate Change
Source: PLoS Biol. 2015 Oct 26;13(10):e1002281. doi: 10.1371/journal.pbio.1002281 (PMC4621050; doi:10.1371/journal.pbio.1002281)
Supplement: S2 Text — (DOCX) [file pbio.1002281.s014.docx]

**Methods:** Temperature, illuminance and hygrometry within the enclosures were recorded every thirty minutes between the beginning of the experiment in June 2012 and the final recapture in May 2014. We calculated mean, maximum and minimum daily (between 10:00 and 18:00) and nightly (between 23:00 and 05:00) temperatures, and averaged them weekly. We also recorded the number of hours per day over the lizards’ critical thermal maximum (CTMax = 40 °C, [1]). Finally, we calculated mean daily illuminance and hygrometry averaged by week. We chose to use parameters recorded between 10:00 and 18:00 for our daily estimates as lizards were rarely active outside of this period in the Metatron. We particularly focused on daily maximum temperatures following Huey’s [2] recommendation about the description of reptiles’ thermal environment. We separated the data into two periods: summer period, between mid-June and the recapture in mid-September, and winter and spring period, between January and mid-May. The summer period is particularly interesting at it corresponds to the months of peak activity in the common lizard [3]; moreover we expected our treatments to be effective mostly during the summer. We first tested whether our ‘intermediate climate’ and ‘warm climate’ treatments resulted in differences between temperatures during the summer. Because these two groups did not differ (F_1,163_ = 1.39, p = 0.24, F_1,163_ = 2.08, p = 0.12, and F_1,163_ = 0.003, p = 0.89, respectively for maximum, mean and minimum summer daily temperatures), we then pooled these two groups together for the subsequent analysis.

**Results:** Over the summer period, mean and maximum daily temperatures differed significantly between ‘present’ and ‘warm’ climate treatments (S1 Table, S1 Fig and S2 Fig). ‘Present climate’ (PC) enclosures showed similar maximum daily temperatures to outside (august maximum daily temperatures in the nearby meteorological station of Saint-Girons-Antichan: 28.1°C) and to lizard populations of origin (august maximum daily temperatures in Villefort meteorological station 29.0°C), while ‘warm climate’ (WC) enclosures were 2°C to 3°C warmer (mean and maximum temperatures respectively, S1 Table, S1 Fig and S2 Fig). The number of hours above the critical thermal maximum differed significantly between treatments (S1 Table). However the difference was likely to be biologically meaningless as temperature during the summer seldom attained 40°C, with an average of only 3.6 minutes per day above CTMax in ‘warm climate’ enclosures. Another notable difference between treatments was the degree of illuminance (higher in WC treatments; S1 Table), and of hygrometry (higher in present climate treatment, S1 Table). It is noteworthy that the variance in daily temperature was higher in ‘warm climate’ enclosures (S1 Table), suggesting that lizards in warmer conditions may experience a greater variation in thermal microhabitat over the day. Indeed, while maximum daily temperatures varied greatly between treatments, minimum daily temperatures were not significantly different (S1 Table). Such low minimum daily temperatures should allow the lizards to evade higher temperatures by shifting their activity timing during the day. Combined with the relatively wide variety of microhabitats available for the lizards inside of the enclosures (shaded vegetation, sun-battered rocks and logs, ponds, Fig 1), this should allow lizards in warmer conditions to avoid extreme temperatures. To verify this assumption, we checked within-enclosure variability in temperatures by placing five thermocouples at different positions inside of the enclosures (near the water, inside of the vegetation, in the air, on the rocks and below the rocks) to record temperatures for a week. We found that temperature varied greatly inside of the enclosures (range of mean daily temperatures between positions: 10.0 ± 1.3 °C, 12.9 ± 1.2 °C, respectively for ‘present’ and ‘warm’ climates). This variation was slightly larger in warm climate (F_1,8_ = 13.6, p = 0.007) because of decreased maximum temperatures in present climate*.* Moreover, while mean temperatures recorded in sun-battered zones differed highly between ‘warm climate’ and ‘present climate’, with a 4.6°C difference in mean temperature recorded at the highest temperature zone of the enclosures (F_1,8_ = 30.3, p < 0.001), differences in lowest temperature zones were much lower, only 1.6 °C (F_1,8_ = 13.9, p = 0.006)*.* Moreover, coolest places in enclosures of all climates had mean daily temperature below 23°C and maximum daily temperature below 26°C (mean daily temperatures: 21.0 ± 0.6 °C and 22.7 ± 0.9 °C, maximum daily temperatures: 22.8 ± 0.9 °C and 25.5 ± 1 °C, respectively for ‘present’ and ‘warm’ climates), which are much lower than lizards thermal optimum (30-34°C, [1,4]), critical maximum temperature (40.4°C, [1,4]) and lower limits of the 80% of thermal performance breath (28.4°C, [1]).

Finally, mean nightly temperatures did not differ between treatments (S1 Table). Over the winter and spring period, and there were no differences in mean and minimum temperature or in hygrometry, but there was a tendency for a higher maximum daily temperature and a significant difference in illuminance between enclosures (S2 Table).

**References**

1. Van Damme R, Bauwens D, Verheyen RF. The Thermal Dependence of Feeding Behaviour, Food Consumption and Gut-Passage Time in the Lizard Lacerta vivipara Jacquin. Funct Ecol. 1991;5: 507. doi:10.2307/2389633

2. Huey RB. Temperature, physiology, and the ecology of reptiles. Biology of Reptilia. Academic Press, London. Gans C, Pough FH; 1982. pp. 25–91.

3. Lepetz V, Massot M, Chaine AS, Clobert J. Climate warming and the evolution of morphotypes in a reptile. Glob Change Biol. 2009;15: 454–466. doi:10.1111/j.1365-2486.2008.01761.x

4. Van Damme R, Bauwens D, Verheyen RF. Evolutionary Rigidity of Thermal Physiology: The Case of the Cool Temperate Lizard  *Lacerta vivipara* . Oikos. 1990;57: 61. doi:10.2307/3565737
