# Supplementary material for: Live Fast, Die Young: Experimental Evidence of Population Extinction Risk due to Climate Change
Source: PLoS Biol. 2015 Oct 26;13(10):e1002281. doi: 10.1371/journal.pbio.1002281 (PMC4621050; doi:10.1371/journal.pbio.1002281)
Supplement: S3 Text — (DOCX) [file pbio.1002281.s015.docx]

**Methods**

We modeled population dynamics in the ‘warm climate’ and in the ‘present climate’ environments using one-sex three age-classes matrix deterministic and stochastic models (Fig. 1). Demographic parameters of the model included primary sex-ratio σ (set at σ = 0.5), juvenile, yearling and adult annual survival probabilities, yearling and adult probability of gravidity (probability to lay eggs) and fecundity (number of viable offspring per gravid female, Fig 1). All demographic parameters were drawn from our experiment using the predicted values of the models exploring juvenile and adult survival, probability of gravidity and fecundity as a function of temperature treatment plus covariates and random effects (see Table 1 for model description, S4 Table for parameter values), except the primary sex-ratio which was set at σ = 0.5, matching observed values in the wild and in our experiment. Initial population sizes were set as the total number of juveniles, yearlings and adult females at the beginning of the experiment in 2012, that is, respectively, 163, 101 and 140 individuals.

We studied the impact of climatic conditions on population growth rate using Legendre and Clobert's Unified Life Model (ULM) software [1]. This software allowed us to obtain population growth rates λ as well as 95 % confidence intervals on λ accounting for the uncertainty in parameter estimates (S4 Table, SE) with the "confidence interval 2" function. We also checked for sensitivity and elasticity of λ to each demographic parameter using the sensitivity function. Finally, we derived time to extinction from the initial population size and λ, considering that a population had become extinct when the number of individuals was lower than 1.

We investigated the impact of demographic parameters on population growth rate in a multistep approach. We first used a deterministic model, using demographic parameters indicated in the S4 Table (results given in result section). In a second step, we checked whether considering females second clutches in warm climates could modify population growth rate. Because of the very low number of offspring produced during this second reproductive event, there was a large uncertainty on the parameter estimates of female summer fecundity and offspring survival. Hence, we decided to add up female summer fecundity (f_summer_ = 0.222 juveniles per females) to the spring fecundity to use this overall fecundity in the matrix model (S4 Table). Finally, we added environmental stochasticity by allowing juvenile, yearling and adult survival parameters to vary between time steps by drawing them from a beta distribution with a mean corresponding to the mean value of the parameter (S4 Table) and a standard deviation of 0.10 with the *beta1f* function in ULM. Such stochastic matrix models allowed taking into account inter-annual environmental variation, as temperature is expected to vary between years. We ran Monte Carlo simulations for 1000 trajectories over 200 time steps using the Monte Carlo option in ULM.

**Results**

Our deterministic model showed that population growth rate in ‘warm climate’ environments was very low (λ = 0.75 [0.72, 0.77], mean [95% CI]), while populations in ‘present climate’ environments were maintaining themselves (λ = 0.98 [0.95,1.01]). Population growth rate was the most sensitive to juvenile and adult survival, and the least sensitive to yearling fecundity (S5 Table). Time to population extinction was different between treatments (years to extinction, mean [95% CI], warm climate = 22 years [20,24], present climate = 298 years [118, no extinction]). In a second step, we added summer second clutches observed in warm climates to female fecundity. This addition did not substantially change the estimated growth rate of the warm climate populations (λ = 0.756 [0.730,0.782]). Finally, although results from our stochastic model were similar to results from our determinist model (Stochastic growth rate α = 0.9697 and α = 0.7296; Mean growth rate λ = 0.984 [0.983,0.985] and λ = 0.730 [0.729,0.731] respectively for present and warm climate), the outcome in terms of extinction risk was quite different, as we observed a non-zero extinction rate in both populations from warm climate and present climate (Probability of extinction p = 0.58 and p = 1.0; Time to extinctions, t = 87.5 [82.6,92.4] years and t = 29.9 [29.7,30.1], respectively for present and warm climate).

**References**

1. Legendre S, Clobert J. ULM, a software for conservation and evolutionary biologists. J Appl Stat. 1995;22: 817–834. doi:10.1080/02664769524649
