# Supplementary material for: Live Fast, Die Young: Experimental Evidence of Population Extinction Risk due to Climate Change
Source: PLoS Biol. 2015 Oct 26;13(10):e1002281. doi: 10.1371/journal.pbio.1002281 (PMC4621050; doi:10.1371/journal.pbio.1002281)
Supplement: S4 Text — (DOCX) [file pbio.1002281.s016.docx]

**Methods:** We searched ISI Web of Knowledge for publications on common lizards using *Zootoca vivipara* and the species former name, *Lacerta vivipara* as keywords. We extracted GPS coordinates of 236 populations across Europe. Populations of common lizards can be viviparous or oviparous. Because our study populations belong to the viviparous lineage, and oviparous forms could be affected differentially by climatic conditions, we only kept the 132 populations that were viviparous (S6 Table). Out of them, 42 populations were surveyed by Sinervo et al., who assigned extinction status to them based on field resurveys [1]. We then used ENSEMBLES daily gridded observational dataset for temperature in Europe (E-OBS version 11.0, 0.25 deg. regular grid, [2], <http://www.ecad.eu/download/ensembles/download.php>) to retrieve information on maximum daily temperatures in these populations. Out of the 132 GPS locations, 2 locations had missing temperature data in the E-OBS database hence we could only retrieve temperature date for 130 populations. We retrieved maximum daily temperature data for 2012, at the start of our experiment, and calculated monthly averages of maximum daily temperatures. We calculated average daily maximum temperature by averaging data of the monthly maximum daily temperature over the two warmest months. We mapped this temperature data to our populations. We then attributed each population to a ‘risk profile’ by comparing its maximum temperature to maximum temperatures experienced by lizards in our experiment (95% confidence interval, IC95% = [28.6, 29.8] and IC95% = [31.5, 32.7] respectively for ‘present climate’ and ‘warm climate’ treatment), and calculating a number of degrees of temperature increase from which populations should be at risk. We attributed populations to 6 risk levels, from A to F, with A = temperature between the lower and upper bound of ‘warm climate’ IC95% temperature; B = temperature between the upper bound of the ‘present climate’ IC95% temperature and the lower bound of the ‘warm climate’ IC95% temperature, C = temperature between the lower and upper bound of the ‘present climate’ IC95% temperature, D = temperature between 1°C below the lower bound of the of the ‘present climate’ IC95% and the lower bound of the ‘present climate’ IC95% temperature, E between 1°C and 2°C below the lower bound of the of the ‘present climate’ IC95% temperature, F = temperature more than 2°C below the lower bound of the of the ‘present climate’ IC95% temperature. These levels were then compared to IPCC projections of climate change depending on four greenhouse emission gas scenarios [3], RCP 2.6 (low emission scenario), RCP 4.5 and 6.0 (mid-range emission scenarios) and RCP 8.0 (high emission scenario). These scenarios predict a temperature increase from 0.4 to 2.6 °C by 2050 (projected temperature rise by 2046-2065, RCP 2.6 = [0.4, 1.6], RCP 4.5 = [0.9, 2.0], RCP 6.0 = [0.8,1.8], RCP 8.5 [1.4,2.6], [3]), and from 0.3 to 4.8°C by 2080 (projected temperature rise by 2081-2100, RCP 2.6 = [0.3,1.7], RCP 4.5 = [1.1, 2.6], RCP 6.0 = [1.4, 3.1], RCP 8.5 = [2.6,4.8], [3]). Level A corresponded to an imminent risk as temperatures already experienced should threaten population persistence. Levels B to C corresponded to close to mid-term risk as a temperature increase between 1 and 2°C (consistent with RPC 4.5) would lead them to experience temperatures corresponding to our ‘warm climate’ treatment. Such levels of temperature increase could be rapidly crossed, as projected temperature rises by 2050 for RCP 4.5 and higher are up to 2°C. Level D corresponds to a risk if temperature increases by 3°C (consistent with RCP 6.0), and E will be at risk if temperature increases by 4°C (consistent with RCP 8.5). Finally, level F is a low risk scenario as it is very unlikely that temperature will rise by at least 5°C.

In a second time, we checked for the robustness of our results to changes in the temperature data by using another climatic database, the CRU dataset [4]. Results were very similar between datasets (33 threatened populations (risk levels A-D) with CRU database in 2012, to be compared to 27 populations with E-OBS database). However, because observations with the CRU database seemed less precise (*pers. comm.,* comparison with known MeteoFrance data), we did not include these last results. Finally, we checked whether inter-annual variability in temperature between 2012 and 2013 changed our results, with overall similar results (23 threatened populations (risk levels A-D) in 2013 against 27 in 2012 for E-OBS database, and 33 versus 33 for CRU database, results not included).

**Results:**

Overall, out of the 130 populations, 27 populations, i.e. 21 %, were considered at risk if temperature increased by up to 3°C (risk levels A to D, consistent with RCP 6.0). If we considered a very important warming of 4°C, possible below the RCP 8.5 high emission scenario, 12 more populations were considered at risk (risk level E). If we considered a more conservative 2°C increase in temperature (risk levels A to C, consistent with RCP 4.5), 18 populations, i.e. 14 %, were threatened. Moreover 14 populations should be threatened in the very near future (risk levels A to B). Finally, it is noteworthy that 2 populations corresponded to the imminent risk level. Interestingly, these populations at immediate risk were those which were at the extreme south margin of the distribution, often populations unconnected to the others. Our risk predictions were congruent with other studies. Indeed, when comparing predicted risk profiles among populations surveyed by Sinervo et al. [1] and found to be recently extinct or committed to rapid extinction and populations found to be maintaining themselves, the number of populations in the risks levels A to C was significantly higher in populations committed to extinction than on populations maintaining themselves (Chisquare test on df = 1, χ² = 7.8, p = 0.005), suggesting that climate change was the main driver of these extinctions. It is noteworthy that all of these populations were at the southern margin of the distribution.

**References**

1. Sinervo B, Méndez-de-la-Cruz F, Miles DB, Heulin B, Bastiaans E, Villagrán-Santa Cruz M, et al. Erosion of lizard diversity by climate change and altered thermal niches. Science. 2010;328: 1354–1354. doi:10.1126/science.1184695

2. Haylock MR, Hofstra N, Klein Tank AMG, Klok EJ, Jones PD, New M. A European daily high-resolution gridded data set of surface temperature and precipitation for 1950–2006. J Geophys Res. 2008;113. doi:10.1029/2008JD010201

3. IPCC. Climate change 2013: the physical science basis : Working Group I contribution to the fifth assessment report of the Intergovernmental Panel on Climate Change. Stocker TF, Qin D, Plattner G-K, Tignor MMB, Allen SK, Boschung J, et al., editors. Cambridge, United Kingdom and New York, NY, USA: Cambridge University Press; 2013.

4. Harris I, Jones PD, Osborn TJ, Lister DH. Updated high-resolution grids of monthly climatic observations - the CRU TS3.10 Dataset: UPDATED HIGH-RESOLUTION GRIDS OF MONTHLY CLIMATIC OBSERVATIONS. Int J Climatol. 2014;34: 623–642. doi:10.1002/joc.3711
